# KLF5-mediated Eppk1 expression promotes cell proliferation in cervical cancer via the p38 signaling pathway

**DOI:** 10.1186/s12885-021-08040-y

**Published:** 2021-04-08

**Authors:** Dong Ma, Zhe Pan, Quan Chang, Jin-jin Zhang, Xiao Liu, Na Hua, Guo-Hua Li

**Affiliations:** 1 School of Public Health, North China University of Science and Technology, 21 Bohai Avenue, Caofeidian New City, Tangshan, 063210 China; 2 Department of Infection Control, the Fourth Hospital of Hebei Medical University, No.12 Jian Kang Rd., Shi Jiazhuang, 050011 China; 3 Tangshan Customs, People’s Republic of China, Tangshan, 063200 China; 4 Department of Oncology, the Second Hospital of Tangshan, Tangshan, 063000 China

**Keywords:** Cervical cancer, Eppk1, KLF5, Proliferation, p38 signaling

## Abstract

**Background:**

Epiplakin1 (Eppk1) is part of epidermal growth factor (EGF) signal and takes part in reorganization of cytoskeleton and cell proliferation. However, the role of Eppk1 in cervical cancer (CC) remains unknown.

**Methods:**

To express Eppk1 and KLF5 and their correlation, we used RNA-sequence, RT-qPCR, TCGA database and immunofluorescence staining in vitro and in different pathological cervical tissues. In CC cell lines, we tested adenovirus-mediated over expression or knockdown of KLF5 and siRNA-mediated knockdown of Eppk1 and a suiting assessment of cell proliferation and cell signaling by western blot and CCK8 tests. We studied the mechanism by which KLF5 regulates Eppk1 expression by reporter gene test and chromatin immunoprecipitation test.

**Results:**

Eppk1 expression promoted in CC tissues and cell lines compared with increased KLF5 expression. The results of immunofluorescence staining further showed the increased co-expression of Eppk1 and KLF5 correlated substantially with tumorigenesis in cervical tissues. Overexpression of KLF5 significantly increased Eppk1 expression at transcription and translation levels. Conversely, the knockdown of KLF5 by siRNA against KLF5 decreased Eppk1 expression. Mechanically, KLF5 activated Eppk1 transcription by direct binding to the Eppk1 promoter. Gain- and loss-of-function experiments reported that KLF5 promoted cell proliferation in Hela partly dependent on Eppk1 upregulation. Besides, KLF5-mediated activation of p38 signaling significantly decreased after Eppk1 knockdown compared with decline of proliferation, suggesting that Eppk1 lies upstream of p38 signaling affecting cell proliferation. Finally, Eppk1 expression is positively correlated with tumor size in clinicopathological features of CC.

**Conclusions:**

Eppk1 may be an effective therapeutic target for affecting p38 signaling pathway and cell proliferation in cervical cancer.

**Supplementary Information:**

The online version contains supplementary material available at 10.1186/s12885-021-08040-y.

## Introduction

Cervical cancer (CC) is globally the most common malignancy of the female reproductive and usually affects young women [1]. Clinically, outcomes in treatment and prognosis of this disease remain suboptimal, with long-term progression-free survival and overall survival rates of about 60% [2]. Its pathogenesis remains unclear, while persistent high-risk human papillomavirus (HPV) infection is the main cause of CC, suggesting investigating of prognostic markers and therapeutic targets is crucial [3]. Applying the high-throughput sequencing technologies, such as RNA-sequence, provides an opportunity for searching them and understanding the molecular mechanism and treatment of CC.

Epiplakin1 (Eppk1), a member of the plakin gene family, is a universal cell linker protein originally identified as a 450-kDa human epidermal self-antigen, mainly expressed in liver, small intestine, stomach, salivary glands, esophagus and skin [4, 5]. Plakin protein binds cytoskeleton myofilaments and anchors at the cytoplasmic membrane junction. More importantly, it is a part of EGF signaling and works in cytoskeleton aggregation and proliferation signal transduction of tumor cells [6]. The varying expression of Eppk1 has found in various cancers such as hepatocellular carcinoma [7], bladder urothelial carcinoma [8], CC [9], and Eppk1 has also further studied in pancreatic cancer [10], but the role of Eppk1 in CC remains unknown.

Kruppel-like factors 5 (KLF5) exerts cell biological roles such as cell proliferation, migration and cytoskeleton polymerization by regulating many important target genes [11]. Our previous study found that KLF5 plays a role in cytoskeleton aggregation and proliferation, and plays an important role in the growth of CC [12], suggesting that there may be a link between Eppk1 and KLF5 expression taking part in CC development.

This study mainly detected the role of Eppk1 in cervical tumogenesis and examined whether KLF5 regulating Eppk1 expression affects EFGR-associated p38 signaling, which will provide molecular targets for clinical late treatment and prognosis of CC.

## Materials and methods

### Clinical samples

Collection of cervical biopsy specimens was from September 2014 to September 2017 in the gynaecology and obstetrics of Tangshan Worker Hospital, with 152 cases including the normal cervical 34 cases (control), cervical intraepithelial neoplasia (CIN) 78 cases (including CIN I 31 cases, CIN II-III 47 cases) and cervical squamous cell carcinoma (CSCC) 40 cases. To investigate the basic information of the patients, and the age of the patients, the age of menarche, the number of sexual partners, the number of pregnancies, the number of abortions, smoking and drinking in different groups had no statistical significance (*P* > 0.05). The median age of all participants was 45 and ranged from 25 to 79. After diagnosis, they underwent surgical resection of primary cervical cancer in the Department of Obstetrics and Gynecology of Tangshan workers’ hospital. To classified the histological types and grades of tumors according to WHO. To established the stage of each cancer according to the International Federation of Gynaecology and Obstetrics (FIGO 2000). According to the agency guidelines, all these patients had got informed consent before sample collection, and to approve the study by the Ethics Committee of North China University of Technology in Tangshan, Hebei Province, China.

### Cell culture

To buy Human nor mal cervical epithelial cell (HcerEpic) and human cervical cancer cell lines (HeLa, SiHa and C33A) from Cell Culture Center (Manassas, VA). These cells were cultured in RPMI 1640 media containing 10% fetal bovine serum, 5% carbon dioxide, 37 °C constant temperature incubator, to use logarithmic growth phase cells for the following experiments.

### Adenovirus vector, siRNA and transfection

To make Ad-KLF5 and Ad-GFP as describing previously [13]. To infect Hela cells with an Adenoviral vector (Genechem Co., Ltd). To achieve RNAi-mediated depletion of Eppk1, we transfected cells with siRNA oligos targeting Eppk1 (si-Eppk1) or negative controls (si-Con) (Genechem Co., Ltd). In this process, we also used the Lipofectamine 2000 reagents (Invitrogen; Thermo Fisher Scientific, Inc.) according to the instructions.

### Immunofluorescence staining

To fix All cervical tissue samples in 4% paraformaldehyde solution, to dehydrat the fixed tissue by ethanol, to transparent by xylene, to set in paraffin, and the sections were 4 μm. To run the samples by an indirect immunofluorescence method according to the instructions. To hatch rabbit anti-KLF5 (1:50, GTX103289, GeneTex) and mouse anti-Eppk1 (1:50, sc-87,102, Santa) overnight at 4 °C, Fluorescein labeled fluorescent secondary antibody, Rhodamine labeled fluorescent secondary antibody (KPL) and DAPI (Sigma) nuclear staining. Image Pro-Plus6.0 (Media Cybernetics, Inc., USA) Image analysis software analyzed the fluorescence intensity of Eppk1 and KLF5 expressions.

### Real-time PCR

To reformed total tissue RNA from the cervical tissues preserved in liquid nitrogen according to the instructions of Trizol kit. To use RNA as template, to synthesize cDNA by reverse transcription kit (Invitrogen, USA), and then to use cDNA as template for Real-time PCR. To invoke Glyceraldehyde-3-phosphate dehydrogenase (GAPDH) as internal reference. Apply Primer Premier Software, design upstream and downstream primers respectively: Eppk1 upstream Primer sequences for 5′- GGCCATGCCGATTTAAATGC-3′, The downstream primer sequences for 5′- CAAGCAAAGTCAGTCCAAGC-3′. The upstream primer sequence of GAPDH was 5′- CGTCCCGTAGACAAAATGGT-3′. The downstream primer sequence of GAPDH is 5′- GAGGTCAATGAAGGGGTCG-3′. Expansion Reaction conditions: 95 °C, 2 min, 95 °C, 15 s, 72 °C, 35 s, 40 cycles. To use Formula 2^-△△Ct^ (Ct as the circulating threshold) calculating the mRNA expression of the target gene, and to repeat the experiment 3 times for each group.

### Western blot

To work a RIPA kit (Beyotime Institute of Biotechnology) extracting total proteins from treated cells. To detect the total protein content by the protein test kit (Beyotime Biotechnology). Using SDS-PAGE to separate the protein samples with 8–12% separating gel and every protein sample added in the well was 40 μg. To resolve The separated proteins and electrophoretically to move to the PVDF membranes. After blocking with 5% defatted milk in TBST at room temperature for 1.5 h, to hatch the blots with primary antibodies for a night at 4 °C. Hatchting the secondary antibody (1:2000) at room temperature, washing the membrane, and preparing the film. The β-actin is the internal reference protein. To detect the signals with the ECL system (Pierce) and to quantify by scanning densitometry with the image J analysis. The primary antibodies includes anti-KLF5 antibodies (1:1000, GTX103289, GeneTex), anti-Eppk1 antibodies (1:1000, sc-87,102, Santa), anti-p38 antibodies (1:1000, 14,064–1-AP, Proteintech), p-p38 (1:1000, 14,064–1-AP, Proteintech), AKT (1:1000, 10,176–2-AP, Proteintech), p-AKT (1:1000, 66,444–1-Ig, Proteintech), p-ERK1/2 (1:1000, cat. No.9101, Cell Signaling), ERK (1:1000, 16,443–1-AP, Proteintech) and β-actin (1:5000, 20,536–1-AP, Proteintech).

### Dual luciferase tests

To use Lipofectamine-2000 co-transfecting pGL3-basic (negative control) vector, pGL3-Eppk1promoter, pGL3-Eppk1 promoter mutation, plasmid-expressed KLF5 or empty plasmid. Concentrating of each plasmid was 0.5 μg/well. Meanwhile, to co-transfect PRL-β-actin 10 ng/well as internal reference. At 24 h after transfection, to transfect the cells with the empty plasmid (pcDNA3.1) and plasmid-expressed KLF5 (pcDNA3.1-KLF5). At 48 h after transfection, to measure luciferase activity using the dual-luciferase reporter test system (Promega), to repeat the experiment three times, and to calculate the average value of the data.

### Chromatin immunoprecipitation tests

To perform the chromatin immunoprecipitation test using the HeLa cells following the protocol provided by Abcam (Cambridge, MA, USA). To hatch the diluted DNA- protein complex with an equal amount of anti-Eppk1 antibody or mouse IgG (Santa Cruz) overnight at 4 °C in the presence of herring sperm DNA and protein A/G beads. To purify Chromosomal DNA and examined by RT-qPCR. The PCR primers for the Eppk1 gene promoter to expand the KLF5-binding region was as follows: Forward: 5’TGGGGCCTGGTGGGGGGAAAG 3′; Reverse: 5’GGCCGGCCCCCTCTGACTCA 3′.To divide the samples into three groups: IgG (negative control), Input (positive control) and Ad- KLF5.

### Cell proliferation test

To use CCK-8 test detecting the growth and proliferation of Hela cells. Hela vaccination in 96-well plates, 100 μl per hole cell suspension, after waiting for cell adherent to group training, each group have 3 hole, cultivate 12 h, 24 h, 48 h, 72 h, 96 h after 10 μl per hole to join CCK-8 solution, set a zero cell free hole, incubator to 4 h incubation, enzyme standard instrument determination at 450 nm OD value.

### Statistical analysis

To analyze data provided as meaning ± standard deviation (^−^x ± s) with SPSS13.0 (SPSS, Inc.) and GraphPad Prism 5.0 (GraphPad Software, Inc.). To compare the pathological data collected from the samples by using chi-square test. To perform Contrasts between two groups through an unpaired two-tailed t test. To use one-way ANOVA for multiple comparisons followed by the post hoc Turkey’s test. *P* < 0.05 examines statistically significant.

## Results

### Eppk1 expression significantly increases in CC tissues and cell lines

**Fig. 1:**
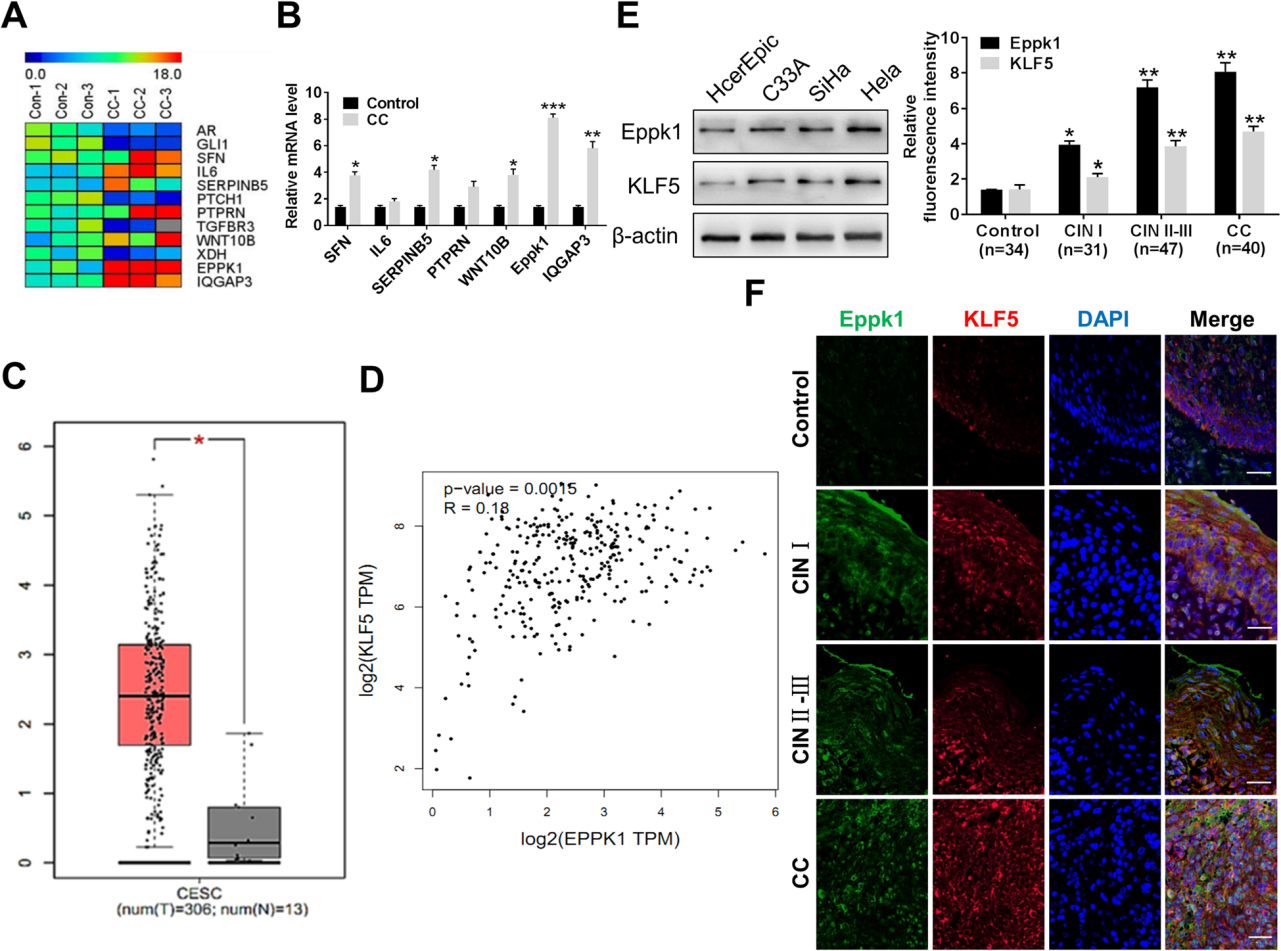
Eppk1 expression increases in cervical cancer tissues and cell lines. **a** Heatmap shows a subset of the differentially expressed mRNAs detected in 3 cervical cancer tissues and 3 normal tissues (control) by micro array analysis. **b** QRT-PCR verifies seven changed genes. ***P < 0.001 vs. control. **c**, **d** mRNA expression of Eppk1 (**c**) and correlation analysis between mRNA expression of Eppk1 and KLF5 (**d**) in cervical squamous cell carcinoma (CSCC) from the Cancer Genome Atlas (TCGA) database. **e** Expression level of Eppk1 and KLF5 protein in C33A, HeLa and SiHa cells. **f** Immunofluorescence staining of KLF5 (red) and Eppk1 (green) in CC tissues (*n* = 40), cervical intraepithelial neoplasia (CIN) tissues (CIN I: 31 cases; CIN II-III: 47 cases) and control (*n* = 37). Scale bars = 100 μm. Right: Statistical analysis of fluorescence intensity. *P < 0.05 and **P < 0.01 vs. control

To examined whether levels of Eppk1 would alter in cervical cancer, we performed RNA expression profiling using RNA-sequence analyses of human CC tissues (*n* = 3) and normal cervical tissues (*n =* 3). As shown in Fig. 1a, mRNA level of Eppk1 was significantly up regulated in CC tissues. RT-qPCR confirmed the increased expression of Eppk1 mRNA in CC tissues was by 8.7 times (*P* < 0.001) compared with the control (Fig. 1b). TCGA database further revealed the level of Eppk1 in cervical squamous cell carcinoma increases (*P* < 0.05) (Fig. 1c). Besides, we also found the correlation between Eppk1 and KLF5 in cervical cancer using the TCGA database (*R* = 0.18, *P* < 0.01) (Fig. 1d). Furthermore, compared with cervical epidermal cells (HcerEpic), the protein levels of both Eppk1 and KLF5 were increased among three CC cell lines (C33A, SiHa and Hela), their expressions were the highest in Hela, which was decided to use this cell line for subsequent experiments (Fig. 1e).

Additionally, we performed immunofluorescence staining to examine co-expression of Eppk1 and KLF5 proteins in different pathologic cervical tissues (37 normal cervical tissues, cervical intraepithelial neoplasia (CIN) tissues (CIN I: 31 cases; CIN II-III: 47 cases) and 40 CC tissues). As shown in Fig. 1f, these results showed that Eppk1 expression markedly increases in CINII-III and SCC tissues with up-regulation of KLF5 expression (both *P* < 0.05 and *P* < 0.01). Also, Eppk1 mainly locates in the intercellular and cytoplasm, suggesting that it mainly acts as membrane receptor and skeleton. These data suggest that Eppk1 up-regulation involves in the tumorigenesis in cervical cancer, which associates positively with KLF5 expression.

### KLF5 is a positive regulator of Eppk1 in CC cell

**Fig. 2:**
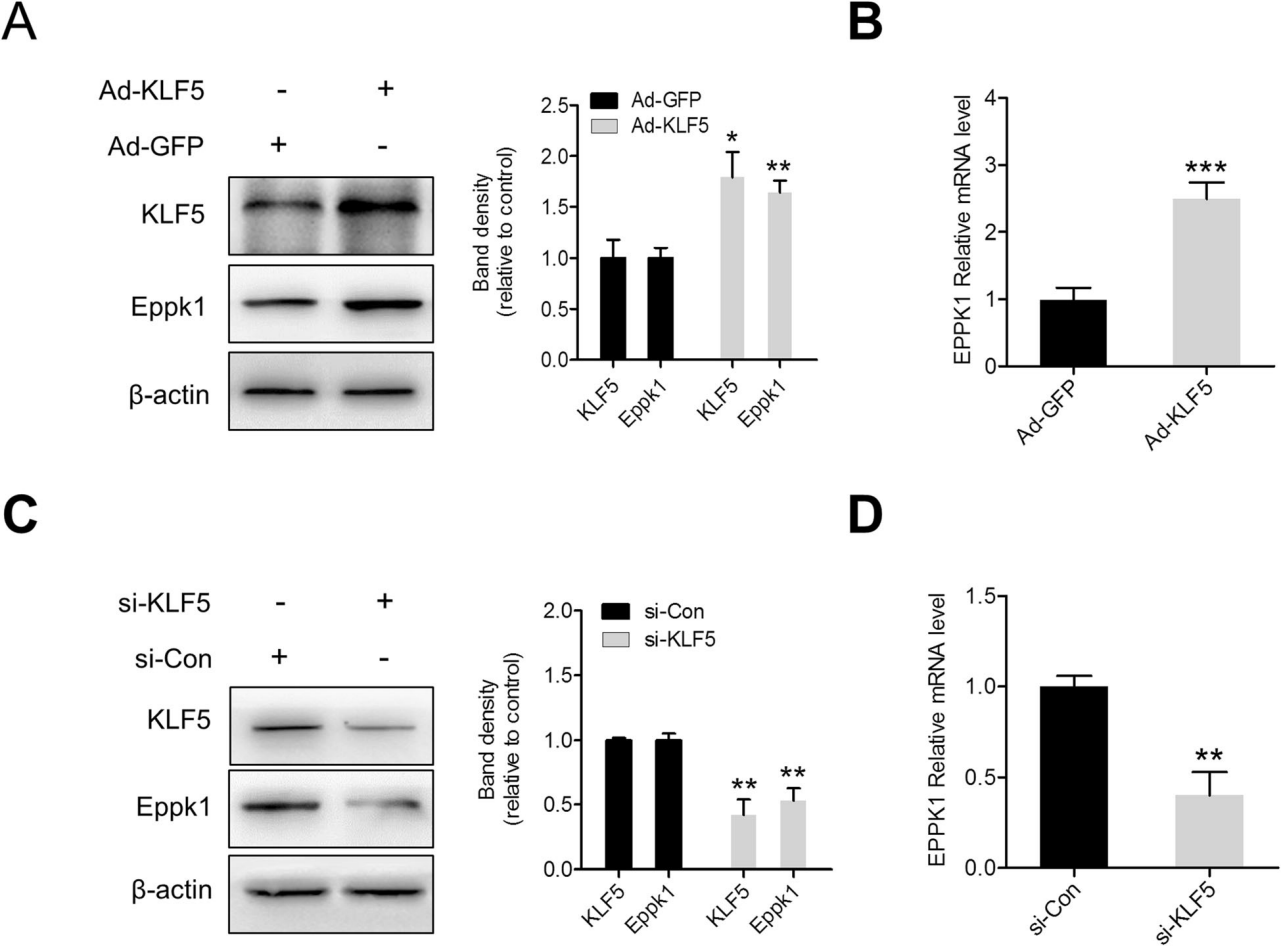
KLF5 is a positive regulator of Eppk1 in Hela cell. **a**, **b** HeLa cells were infected with adenoviruses expressing green fluorescent protein (Ad-GFP) or KLF5 (Ad-KLF5) for 24 h (10 ng/mL). KLF5 and Eppk1 protein and mRNA expression were analyzed by Western blot (**a**) and qRT-PCR (**b**), respectively. *P < 0.05 and **P < 0.01 vs. the Ad-GFP. **c**, **d** HeLa cells were transfected with non-specific short interfering RNA (si-Con) or KLF5-specific siRNA (si-KLF5) for 24 h. KLF5 and Eppk1 protein and mRNA expression was analysed by western blot (**c**) and qRT-PCR (**d**), respectively. *P < 0.05 vs. the si-Con

To further confirm whether KLF5 needs for expressing of Eppk1 gene, we makes KLF5 over expressing and knockdown in Hela cells by infecting with adenoviruses encoding KLF5 (Ad-KLF5) or by transfecting with siRNA against KLF5 (si-KLF5), respectively. Both RT-qPCR and Western blot results showed the over expression of KLF5 increased expression of Eppk1 of transcription and translation levels (Fig. 2a, b). Conversely, knockdown of KLF5 suppresses Eppk1 expression in Hela cells (Fig. 2c, d). These results show that KLF5 may play a part in the regulation of Eppk1 expression.

### KLF5 regulates Eppk1 expression by direct binding to the Eppk1 promoter

**Fig. 3:**
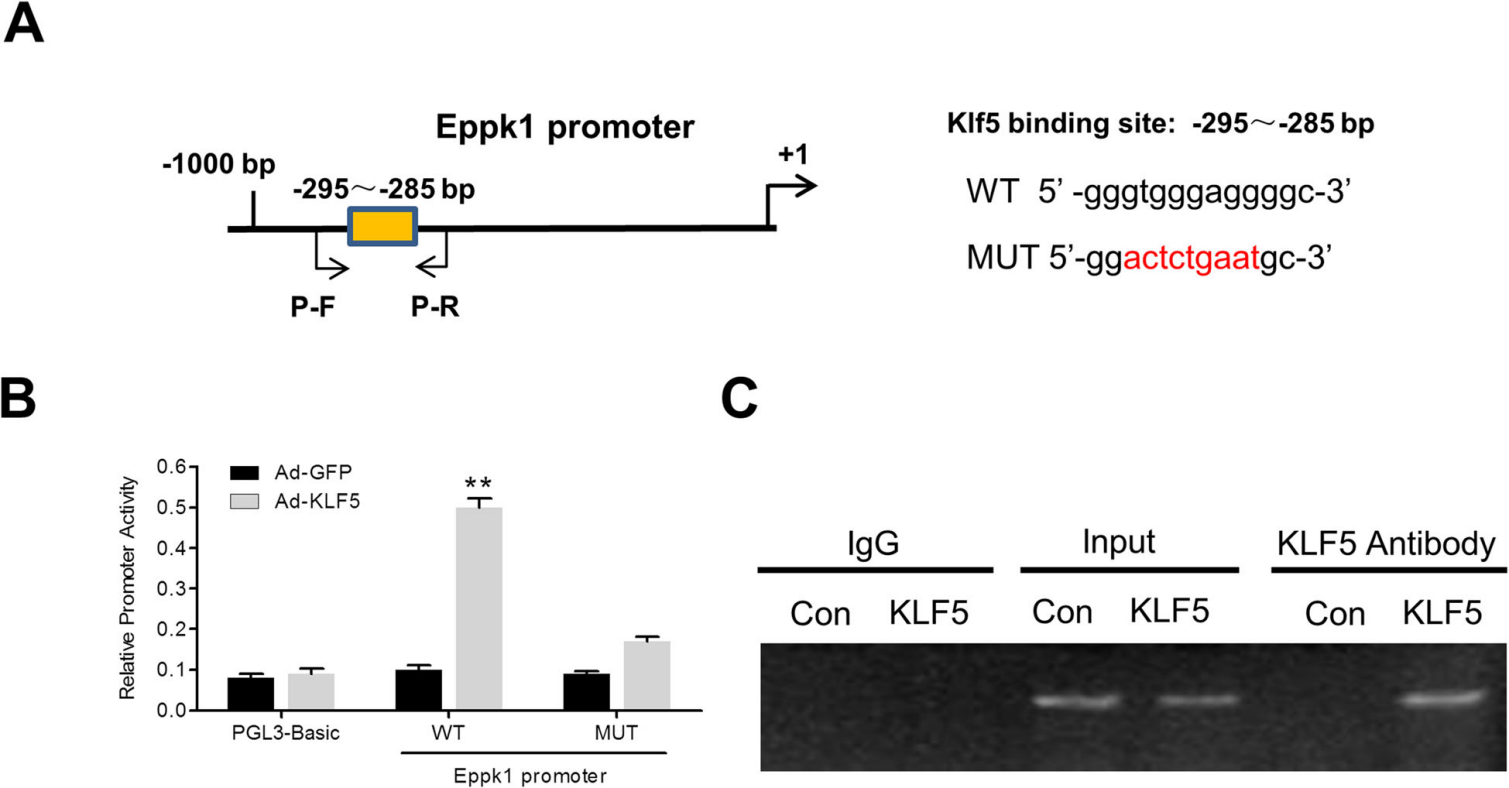
KLF5 regulates Eppk1 expression by direct binding to Eppk1 promoter. **a** A schematic map of the − 1000 to + 1 bp regions of the Eppk1 promoter showing a KLF5-binding site at − 295 to − 285 bp region. **b** A luciferase reporter controlled by the Eppk1 promoter or the Eppk1 promoter with a mutation in KLF5-binding site was transfected into Hela cells with increasing amounts of KLF5 expression plasmid (pcDNA3.1-KLF5). PGL3-Basic was used as the negative control. Luciferase activity was monitored using the dual luciferase reporter test system. Data represent relative Eppk1 promoter activity normalized to pRL-TK activity. **P < 0.05 vs. pcDNA3.1. **c** Hela cells were infected with or without Ad-KLF5 for 24 h. ChIP test was then performed with an antibody against Klf5. Nonimmune IgG was served as negative control. Immunoprecipitated DNA was amplified by PCR using the primers showed as in (**a**)

To prove whether Klf5 regulates the expression of Eppk1 by directly binding to its promoter, using the JASPAR CORE database, we performed a KLF5-binding motif analysis on the Eppk1 promoter, and identified one typical KLF5-binding site in the − 1000 to + 1 bp of the 5′upstream promoter of Eppk1 gene (Fig. 3a). Later, the Eppk1 promoter and its mutant constructs and tests by the luciferase activity test. The results show that enforced KLF5 expression markedly increases in activity of the Eppk1 full-length promoter. Mutation of − 295 to − 285 bp regions of the Eppk1 promoter significantly decreased this activation by KLF5 (*P* < 0.01) (Fig. 3b), showing that KLF5-binding site between − 295 to − 285 are critical for the KLF5-mediated transcriptional activation of the Eppk1 promoter. Moreover, chromatin immunoprecipitation (CHIP) test further proved that over expression of KLF5 obviously increased its recruitment to the site (Fig. 3c). These results suggested that KLF5 activates Eppk1 transcription by directly binding the Eppk1 promoter.

### KLF5 promotes CC cell proliferation dependent on Eppk1 expression

**Fig. 4:**
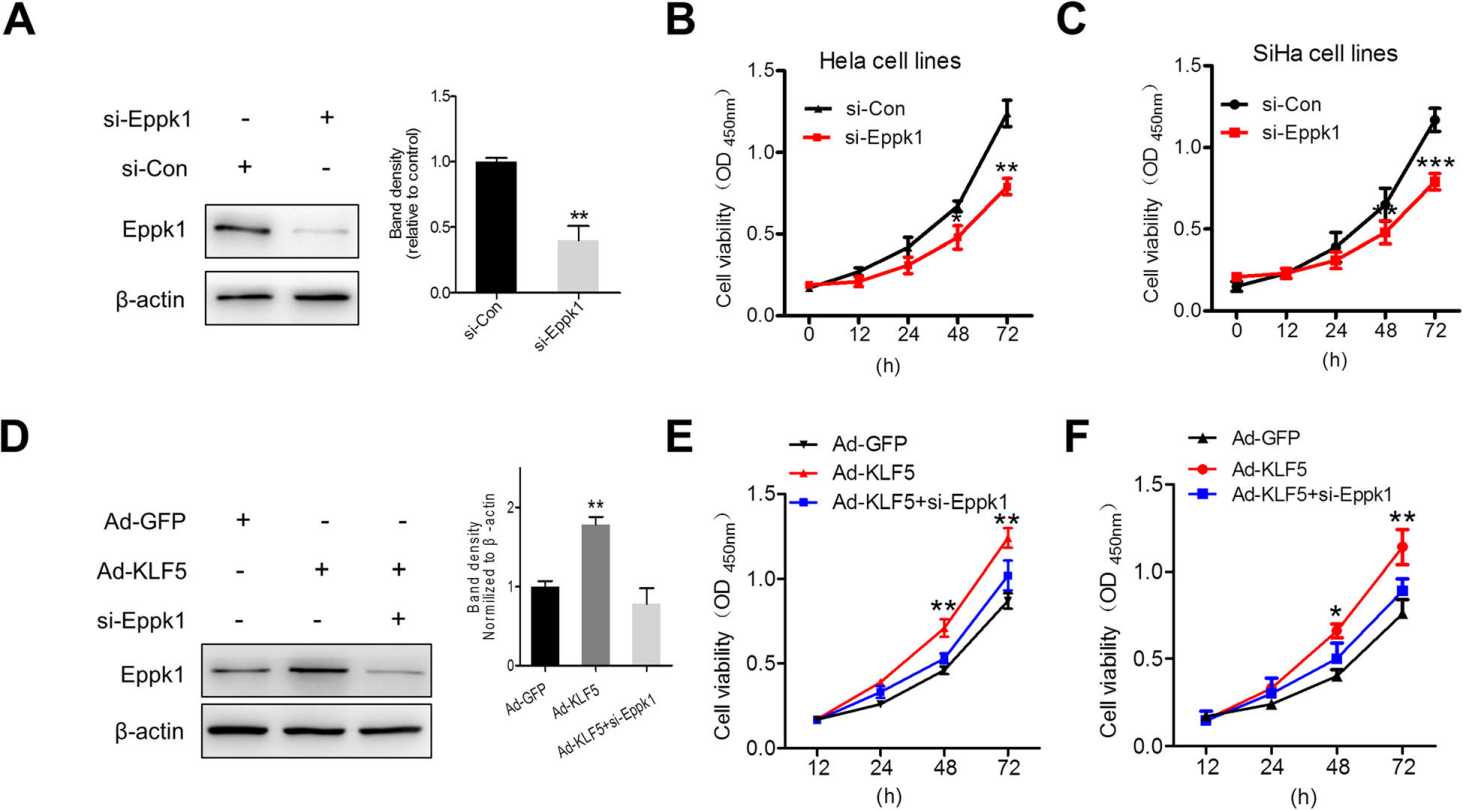
Eppk1 lies downstream of KLF5 and promotes Hela cell proliferation. **a** HeLa cells were transfected with si-Con or Eppk1-specific siRNA (si-Eppk1) for 24 h. Eppk1 protein expression was analysed by western blot. β-actin was used as the loading control. Right: band intensities were measured and normalized to β-actin (*n* = 3). Data represent the mean ± SD. **P < 0.01 vs. si-Con. **b** and **c** Knockdown of Eppk1 significantly affected HeLa (**b**) and SiHa (**c**) cell growth compared to transfection with si-Con at different indicated time points by a CCK-8 test (*n =* 3). *P < 0.05, **P < 0.01 and ***P < 0.001 vs. the si-Con. **d** Ad-GFP and Ad-KLF5-infected HeLa cells and Ad-KLF5-infected HeLa cells were transfected with si-Eppk1 for 36 h. Expression of Eppk1 was confirmed by western blot. Right: band intensities were measured and normalized to β-actin. Data represent the mean ± SD. **P < 0.01 vs. Ad-GFP. **e** and **f** CCK-8 assay for cell proliferation in Hela and SiHa cells treated as in (**b**) and (**c**) respectively. Data represent the mean ± SD. *P < 0.05 and **P < 0.01 vs. Ad-GFP

As indispensable role of transcription factor KLF5 in cell proliferation and tumorigenesis in CC, we determined whether Eppk1 expression can affect cell proliferation. As shown in Fig. 4a, Eppk1 expression markedly decreases in si-Eppk1-transfected HeLa cells. Correspondingly, cell viability significantly was suppressed through CCK8 assay (Fig. 4b). Similarly, cell viability significantly was also suppressed in si-Eppk1-transfected Siha cell lines (Fig. 4c). Further, improved KLF5 expression inducing the up-regulation of Eppk1 expression and cell proliferation were also significantly suppressed in si-Eppk1–transfected Hela and Siha cells (Fig. 4d-f), suggesting that Eppk1 requires for cell proliferation in CC. These results suggested that KLF5 mediates Eppk1 expression promoting CC cell proliferation.

### Eppk1 expression affects p38 signal pathway

**Fig. 5:**
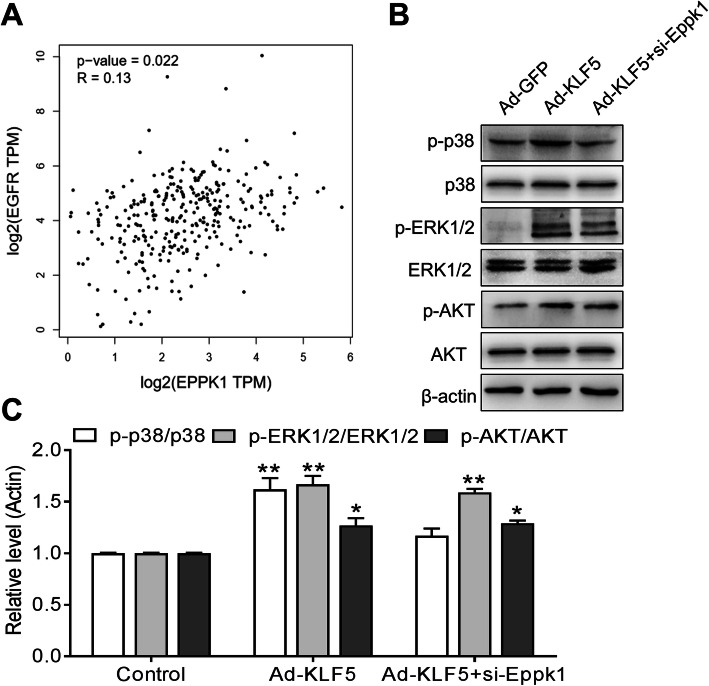
Eppk1 affects EGFR expression and p38 signal pathway. **a** Correlation analysis between mRNA level of Eppk1 and EGFR in CSCC tissues from TCGA database. **b** Ad-GFP and Ad-KLF5-infected HeLa cells and Ad-KLF5-infected HeLa cells were transfected with si-Eppk1 for 36 h. Western blot was used to decide the activation of AKT, ERK1/2 and p38 signaling. **c** Band intensities were measured and normalized to β-actin or total AKT, ERK1/2 and P38 (*n* = 3). *P < 0.05 and **P < 0.01 vs. control, ^#^*P* < 0.05 vs. Ad-GFP

Because Eppk1 is a part of EGFR, and Eppk1 expression has a positive correlation with EGFR in CC from TCGA data (*P* < 0.05, *R* = 0.13) (Fig. 5a), we further clarify the EGFR-related signaling pathways by which Eppk1 expression affects the proliferation of cervical cancer cells. As shown in Fig. 5b, the activation protein of p-p38, p-ERK1/2 and p-AKT all significantly increase (*P* < 0.01) in Ad-KLF5-infected Hela cells, whereas KLF5 expression-induced p-p38 activation significantly decreases after si-Eppk1 transfection (*P* < 0.05), and p-AKT and p-ERK1/2 signaling pathways apparently were not affected (Fig. 5b and c). Also, we found out the correlation between the expression of Eppk1 and clinicopathological features in CC tissues. The results showed the mRNA level of Eppk1 linkes to HPV positive infection rate and tumor size (*P* < 0.05), but not with pathological grade and clinical stage (Table 1). Taken together, these data suggest that KLF5-mediated Eppk1 expression affecting p38 signaling, the KLF5-Eppk1-p38 axis regulates cell proliferation and the tumorigenesis and development of CC.

**Table 1:** The relationship between expression of Eppk1 mRNA and pathological characteristics of CC

Parameter	N	Eppk1 low expression	Eppk1 high expression	*χ*^2^	*P-value*
	40	N(%)	N(%)		
HPV16/18 type				4.42	0.035
positive	29	21(72.4)	8(27.6)		
negative	11	4(36.4)	7(63.6)		
Tumor size(cm)				6.07	0.014
≤ 4	14	10(71.4)	4(28.6)		
>4	26	8(30.8)	18(69.2)		
Pathological grading				2.13	0.145
High and medium differentiation	13	8(61.5)	5(38.5)		
poorly differentiated	27	10(37.0)	17(63.0)		
clinical stages				1.32	0.251
I	23	15(65.2)	8(34.8)		
II	17	8(47.1)	9(52.9)		

## Discussion

The present study firstly shows that Eppk1 plays a crucial role in positively regulating cell proliferation and Eppk1 as a direct target of KLF5. Specifically, we viewed that knockdown of Eppk1 expression blocked KLF5-induced cell proliferation via p38 signaling pathway. Additionally, KLF5 and Eppk1 co-express in human CC tissues and CC cell lines. Our results suggest that Eppk1 and p38 signaling may be the possibility therapeutic targets for the CC treatment.

Most plakins containing a plakin domain formed by up to nine spectrin repeats (SR1–SR9) and a SH3 domain usually expresses in various organs and tissues [14], but detailed expression patterns are still not examined. Eppk1 as a member of plakin family, expresses in various progenitors and developing and regenerating cells, especially in pancreatic cancer [15, 16], suggesting that it could be a good marker for development of cancer cells. In this study, we focused on the expression pattarns of Eppk1 in cervical tissues and found that under-expression of Eppk1 is in normal cervical tissues and slightly increase in CINItissues. However, it significantly increases in severe pathological changes in cervical tissues, such as CINII-III and cancer, which would be like to the regeneration and development of injured tissues or the tumorigenesis and development of cancer with upregulation of Eppk1. It hypothesizes that some cancers represent an aberrant summary of normal progress, even that further deduction that some cancers originate from adult tissue stem cell [17]. Besides, expression of Eppk1 was a positive correlation with tumor size in CC tissues (Table 1). Therefore, up-regulation of Eppk1 expression might shed light on initiation and development of cervical cancer. In addition, Marrero-rodriguez et al. found that KLF5 showed a gradual up-regulation trend in normal tissues, low-grade intraepithelial lesions, intraepithelial squamous lesions and CC samples, showing the up-regulation of KLF5 expression plays a certain role in the case and development of cervical cancer [18]. Similarly, we also proved the co-expression pattern between KLF5 and Eppk1 and Eppk1 is a target of KLF5 in CC cells, verified that restraint of KLF5 expression by pharmacologic treatment (such as ML264) may be good for treatment of CC.

It knowns that promoted levels of the epidermal growth factor receptor (EGFR), a growth factor receptor tyrosine kinase, and/or its cognate ligands have identified as a common part of multiple cancer types and promote solid tumor growth [19]. When EGFR triggers by growth factors, it mainly regulates cell proliferation and other biological processes through activation of downstream signaling pathways [20]. EGFR activation-associated downstream signaling pathway mainly includes ERK [21], p38 [22] and AKT [23] signaling pathways, which have to explore on the potential functional mechanism by which Eppk1 mediates cell proliferation of CC. Besides, KLF5 widely expresses in different tissues and plays a higher role in cell proliferation than in cell differentiation [24]. It has noted that KLF5 is a positive regulator of the RAS/MAPK signaling pathways, and the RAS/MAPK pathway is affected by KLF5-mediated EGFR activation [25, 26]. Our previous study also revealed the p38 signaling pathway mediated the effects of KLF5 in CC cell proliferation [12]. In addition, Eppk1-positive cells express PCNA (a proliferation marker), which plays an important role in transient amplification of cells [27], and Blagoev and others found that plakin identifies as one of the proteins binding to EGF receptor using proteomics, suggesting that Eppk1 involves in EGFR-related proliferation signal transduction in tumor cells [6]. In this study, we found the expression of Eppk1 is positively correlation with EGFR expression from TCGA database, simultaneously restraint of p38 phosphorylation by Eppk1 knockdown suggest that cell proliferation of CC partly dependent on p38 signaling pathway via Eppk1 expression in context of KLF5 expression, but not the ERK or AKT signaling pathway. Therefore, restraint of Eppk1 expression or its downstream p38 signaling pathway may exert useful effects for CC treatment. A major limit of the present study is the role of Eppk1 in CC cell growth should verify in the body; secondly, some other biological functions of Eppk1 in CC cells still need to assess. Last, Eppk1 lack in CC model causing a phenotype of tumor growth restraint is also essential. Therefore, treatment of CC with restraint of Eppk1 and/or p-38 signaling can be extra to other interventions for the development of the disease.

## Conclusions

Collectively, our studies discovered that KLF5-mediated upregulation of Eppk1 can activate the p38 signaling pathway to promote the proliferation of CC cells. This work excites attention on the role of Eppk1 in CC and gives the direction for future research, contributing to a novel molecular target for the clinical diagnosis and treatment of cervical cancer.

## Supplementary Information

**Additional file 1.**

## Data Availability

All data produced or analysed during this study are included in this published article.

## Abbreviations

Eppk1: Epiplakin1
HPV: Human papillomavirus
KLF5: Kruppel-like factors 5
CIN: Cervical intraepithelial neoplasia
SCC: Squamous cell carcinomas
FIGO: Federation of Gynaecology and Obstetrics
HcerEpic: Human normal cervical epithelial cell
DAPI: 2-(4-Amidinophenyl)-6-indolecarbamidine dihydrochloride
GAPDH: Glyceraldehyde-3-phosphate dehydrogenase
Ct: The circulating threshold
Ad-KLF5: Adenoviruses encoding KLF5
si-KLF5: siRNA against KLF5
CHIP: Chromatin immunoprecipitation
EGFR: The epidermal growth factor receptor
ERK: Extracellular signal-regulated kinase
MAPK: Mitogen-activated protein kinase
PCNA: Proliferating Cell Nuclear Antigen
