# Supplementary figures and images for: KLF5-mediated Eppk1 expression promotes cell proliferation in cervical cancer via the p38 signaling pathway
Source: BMC Cancer. 2021 Apr 8;21:377. doi: 10.1186/s12885-021-08040-y (PMC8028205; doi:10.1186/s12885-021-08040-y)

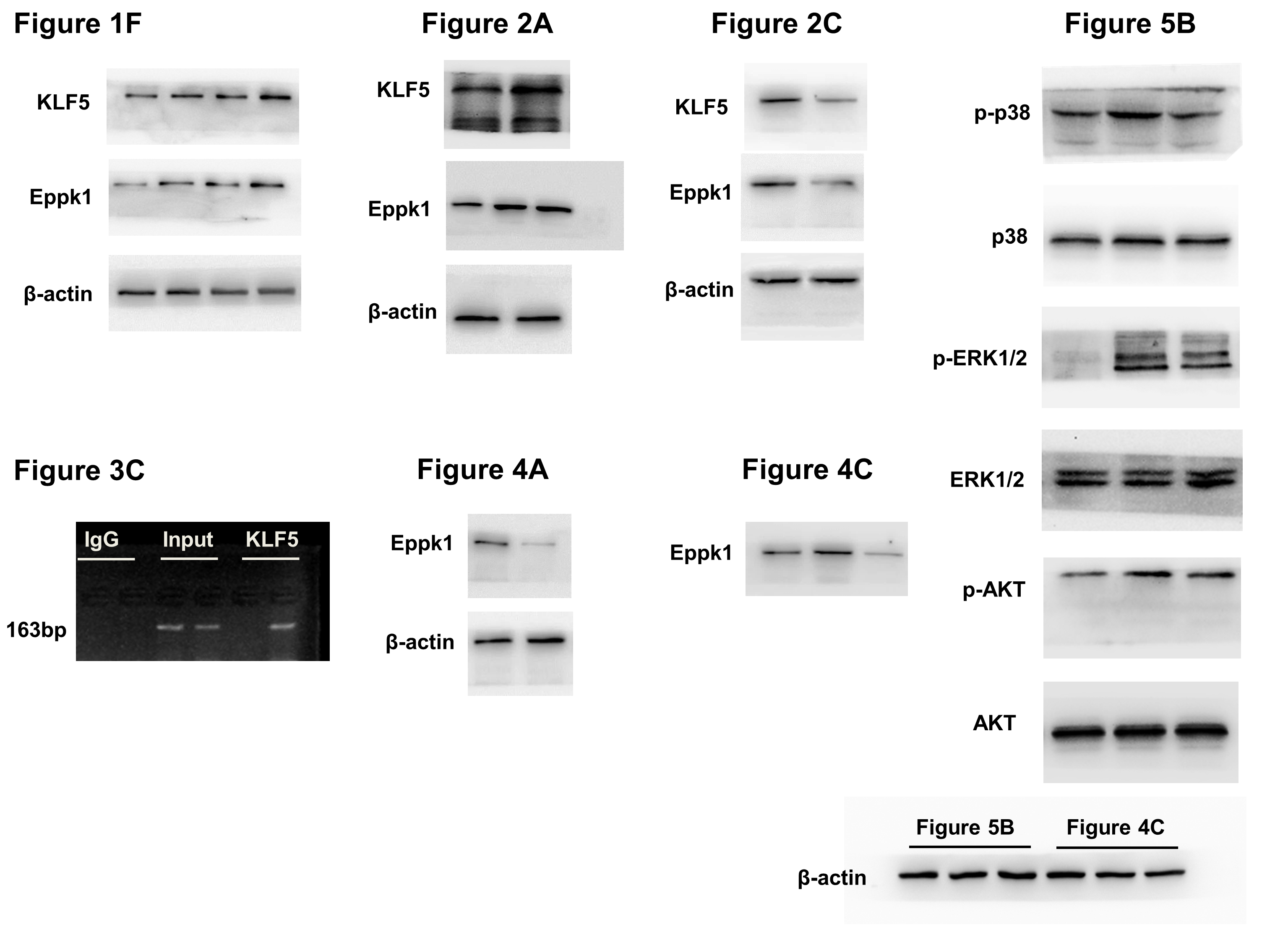

Supplement: Supplementary file 1 — Additional file 1. [file 12885_2021_8040_MOESM1_ESM.tif]
